# Management of cutaneous tuberculosis in hand – Rare and disabling: A case report

**DOI:** 10.1016/j.ijscr.2024.109631

**Published:** 2024-04-05

**Authors:** Hardisiswo Soedjana, Betha Egih Riestiano, Lisa Y. Hasibuan, Selvy Harianti

**Affiliations:** Division of Plastic Reconstructive and Aesthetic Surgery, Department of Surgery, Faculty of Medicine, Universitas Padjadjaran, Bandung, Indonesia

**Keywords:** Cutaneous tuberculosis, Necrotomy debridement, Tissue biopsy

## Abstract

**Introduction and importance:**

Cutaneous Tuberculosis (CTB), elicited by the *Mycobacterium tuberculosis* complex, manifests dermatologically. The scarcity of bacilli within CTB lesions renders their detection challenging. This study presents a case of CTB, underscoring its rarity and the potential for severe complications that can deteriorate patient quality of life. It aims to highlight the importance of CTB identification in dermatological diagnoses due to its capacity to cause considerable morbidity and affect patients' psychosocial health.

**Case presentation:**

An 18-year-old patient presented with a painful, well-defined reddish plaque on the right palm, originating five years prior, accompanied by contractures of the middle finger. The tender lesion, characterized by an irregular surface, exhibited purulent discharge upon light touch through fissures along its periphery. Management involved necrotomy, debridement, and tissue biopsy for diagnostic and reconstructive purposes.

**Clinical discussion:**

CTB exhibits a wide range of clinical presentations, often resembling other dermatological infections, which complicates its diagnosis. Accurate diagnosis necessitates an integrated approach involving clinical assessment, the tuberculin skin test, histopathological analysis, and bacteriological investigations. The therapeutic regimen includes multidrug anti-tuberculosis treatment, with surgical intervention reserved for specific cases.

**Conclusion:**

Long-term complications of untreated CTB encompass significant contractures, scarring, and the onset of carcinomas and sarcomas. Prompt diagnosis facilitates timely and effective treatment, averting these sequelae and yielding high patient satisfaction.

## Highlights

• Tuberculosis (TB) remains a significant global health issue, with millions of new cases and deaths reported annually
• Cutaneous tuberculosis poses a serious threat in those with high prevalence
• Early diagnosis results in appropriate treatment, preventing the sequelae events and high levels of patient satisfaction.

## 1 Introduction

Tuberculosis (TB), a disease as ancient as civilization itself, remains a critical public health challenge worldwide, ranking as the second leading cause of death from a single infectious agent [1]. In 2021, TB was responsible for 10.6 million new cases and 1.6 million deaths, highlighting its significant impact, particularly in low- and middle-income countries which bear the brunt of the disease [2]. The disproportionate distribution of TB cases, notably in the WHO regions of South-East Asia and Africa, underscores the need for targeted interventions in these high-burden areas [3].

Extra-pulmonary TB (EPTB), making up 22 % of TB cases in Europe, demonstrates the disease's ability to affect various anatomical sites beyond the lungs, such as the lymph nodes, urogenital tract, and skin.4 Among these, cutaneous TB (CTB) is a rare but significant form, especially challenging to diagnose due to its diverse manifestations and the commonality of immunocompromised patients among its demographic.6,7 The interplay between TB and factors like the HIV epidemic, migration, and crowded living conditions has exacerbated the resurgence of TB, complicating efforts to control its spread [[1], [2], [3], [4]].

The diagnosis of CTB remains difficult, often requiring a multidisciplinary approach to manage the broad spectrum of its presentations effectively. Despite advancements in diagnostics, the disease's mimicry of other conditions and the reduced sensitivity of tests for cutaneous forms demand comprehensive strategies to confirm cases [5,8,9]. Ensuring accurate diagnosis is crucial, necessitating a combination of histopathological analysis, tuberculin skin tests, chest radiographs, and molecular techniques like PCR to improve disease management and patient outcomes [9].

This streamlined introduction aims to capture the essence of the original text, focusing on the epidemiology, significance, and challenges of TB and CTB while maintaining the cited references to preserve the scientific credibility of the information presented. Additionally, the patient in this case report was provided with informed consent regarding potential publication and this work has been reported in line with the SCARE criteria [10].

## 2 Case report

**Fig. 1:**
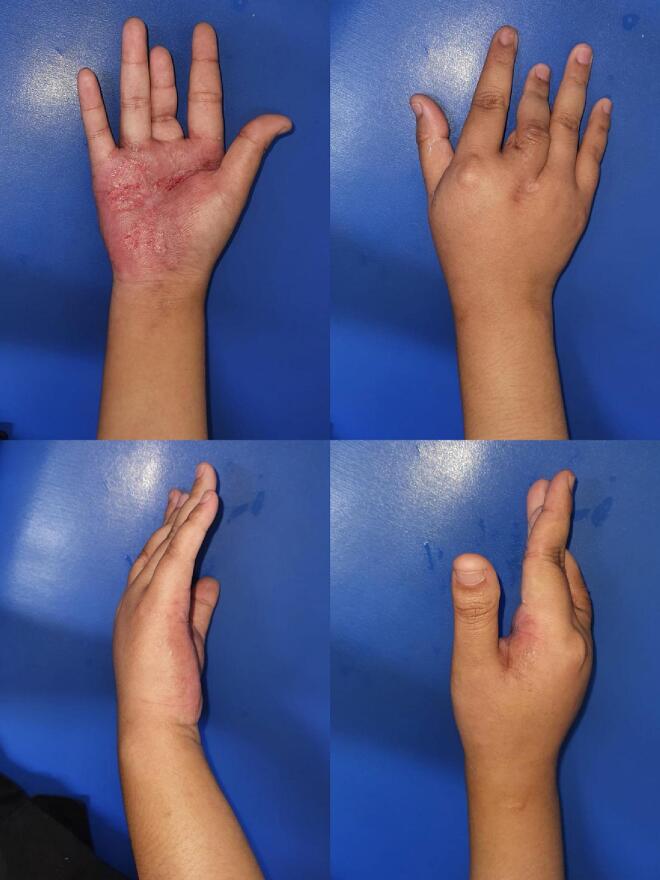
Erythematous scaly plaques on her right palm.

18-year-old Indonesian woman, presenting with a complex medical history and challenging diagnostic journey. Initially presenting with a painful lesion on the right palm, which developed following a traumatic injury and exhibited characteristics such as reddish swelling, well-defined borders, contractures of the middle finger, and discharge of purulent material, the patient's condition did not align with typical diagnoses of cellulitis or chronic osteomyelitis based on her history and clinical findings (Fig. 1).

The absence of common predisposing factors for cellulitis, alongside negative indicators for chronic osteomyelitis such as fistulous tracts, acute musculoskeletal pain, or constitutional symptoms, prompted further investigation. The clinical examination revealed an erythematous, edematous scaly plaque on the right palm, with features not entirely consistent with the initially considered differential diagnoses.

**Fig. 2:**
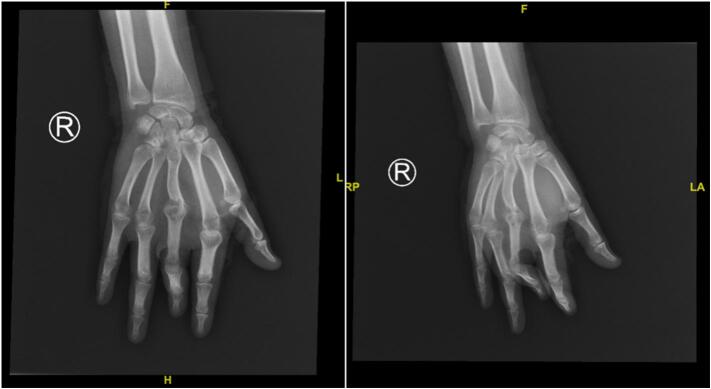
Anteroposterior and Oblique X-Ray of Hand.

**Fig. 3:**
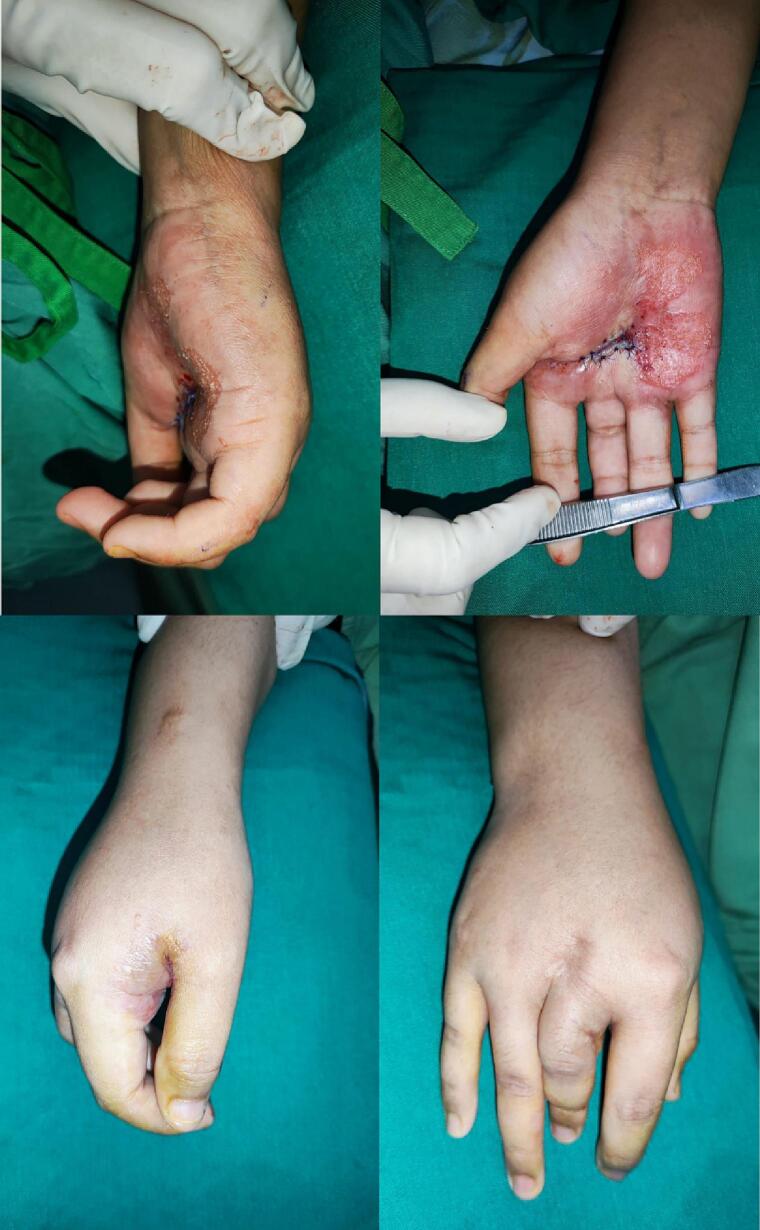
Her right palm after the surgery.

The diagnostic process included routine investigations, chest X-ray, and specific imaging of the right palm (Fig. 2), alongside a positive tuberculin skin test indicating TB exposure. From the anamnesis, no history of TB exposure was found in the home or school environment. Surgical intervention comprising necrotomy, debridement, and contracture release, followed by skin flap, was undertaken (Fig. 3). Histopathological examination of the biopsy revealed pseudoepitheliomatous hyperplasia and non-caseating granulomas, leading to a diagnosis of CTB, potentially lupus vulgaris (LV) or tuberculosis verrucosa cutis (TVC).

**Fig. 4:**
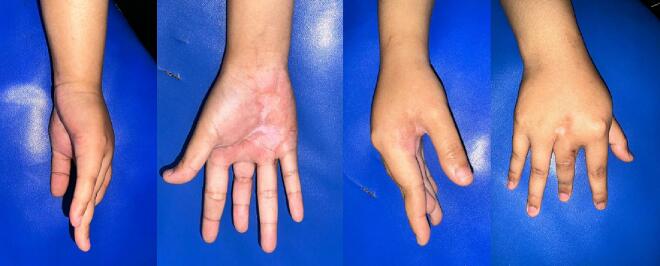
Post operative lesion showed significant improvement.

The patient's subsequent improvement under multidrug TB therapy, consistent with WHO recommendations for our country using a Fixed Drug Combination (FDC) of 150 mg rifampicin, 75 mg isoniazid, 400 mg pyrazinamide, and 275 mg ethambutol, underscores the importance of considering CTB in the differential diagnosis of persistent, non-healing cutaneous lesions. This is especially critical in endemic regions or in patients with a history suggestive of TB exposure. A month after surgery and TB therapy, the wound went well (Fig. 4).

## 3 Discussion

The bacteria that cause the infectious and contagious disease TB is known as *Mycobacterium tuberculosis*. The more uncommon form of this infectious disease, CTB, accounting for 1 to 1.5 % of all extrapulmonary TB manifestations, is prevalent only in 8.4–13.7 % of all TB cases [11]. True CTB, or TB infection, can be further defined as either an unusual mycobacterium species or an infection from *M. tuberculosis*. The host's immunity, the site of the infection, or even the surrounding environment of the host are several of the external factors that can affect how the disease appears clinically [7].

*Mycobacterium tuberculosis* is a straight or slightly bent (rod-shaped), nonmotile, non-sporulated, bacillus, being 1 to 10 nm long and 0.2 to 0.6 mmm wide; its most important feature is acid-fastness due to high lipid content in the cell wall. Approximately there are 4000 genes with most of them involved in the mechanism of immune system invasion and 200 of them for lipid metabolism; consequently, the pathogen is able to survive both inside and outside the phagocytic cells [11]. Meanwhile, as lipids are the main energy source of *Mycobacterium tuberculosis*, the pathogen is directly responsible for multiplying in host tissue and forming cellular walls [11,12]. In this presenting case we could not implemented bacterial culture however skin histopathological examination revealed inflammation which characterized TB infection.

The CTB immune spectrum is widely known and initially proposed by Sehgal et al. as a continuum that extends from the pole of greater activity in cellular immunity—as observed in lupus vulgaris, active cellular immunity (and apparently normal levels of immunoglobulins)—to the pole of less activity in cellular immunity, although with high humoral response, as in the case of scrofuloderma and milliary TB [13,14].

Introducing more specific and sensitive diagnostic methods and understanding better the cellular and molecular mechanisms that regulate the host-agent interaction may contribute to an efficient control of TB. The immunosuppression represents the main trigger for active TB [15].

Macrophages are one of the first lines of defense against mycobacteria. After being phagocytosed, the bacilli remain within the phagosome. Later, the antigens can be processed and presented to CD4 + lymphocytes through class II major histocompatibility complex, and subsequently stimulate the CD8+ T cells [16,17].

Because CTB is uncommon and can present with a variety of morphologies, it often goes unnoticed. In addition to histology, culture, and PCR, a strong index of suspicion is necessary for the diagnosis of CTB Systemic involvement is found in about one-third of CTB cases. According to their mode of dissemination, CTB symptoms are usually classified as exogenous, endogenous, or hematogenous [18]. TB verrucosa cutis and tuberculous chancre are examples of exogenous CTB. TB orificialis and scrofuloderma are examples of endogenous CTB. Acute miliary TB, metastatic tuberculous abscess, and lupus vulgaris are examples of hematogenous dissemination that results in CTB. When a cutaneous lesion fulfills any of the above features, doctors should be willing to consider CTB as a differential diagnosis [19]. In this case, formerly we also overlooked the diagnosis of CTB due to its rarity even in endemic TB country.

Tuberculous chancre or tuberculosis verrucosa cutis are the symptoms of an exogenous primary inoculation of cutaneous TB, which is caused by *M. tuberculosis* penetrating the skin and mucosa. The combination of tuberculous chancre and regional adenopathy may function as the cutaneous analog of the Ghon complex, which is a crucial discovery of primary pulmonary TB [20]. These lesions usually develop following a local trauma, such as tattoos or unsterilized surgical techniques. Initially, a reddish-brown, firm, nontender papulonodular lesion with enlarged lymph nodes is the appearance of a tuberculous chancre. The lesion expands quickly, then begins to erode, leaving behind a well-defined, painless ulceration with red, blue, and weakened margins and a coarse, granular base [20]. In this presenting case *M. tuberculosis* also postulated got the entry through exogenous inoculation. Her wound after local injury may become infected by these bacteria after inappropriate management.

These lesions can last for many years, but they can sometimes spontaneously disappear without treatment, leaving behind atrophic scars, or develop into a lesion that resembles lupus vulgaris. The disease moves from the lungs to the skin and mucosa as a result of post-primary, or secondary, exogenous CTB inoculation. Post-primary inoculation CTB, or TB verrucosa cutis, is a reddish-brown papule that grows slowly and doesn't show any lymphadenopathy. It grows into a verrucous plaque with clefts and fissures on the surface that resembles a common wart. Usually, an extremity—like the hand or foot—is affected. Progression is characterized by the development of many warts, papules, and sensitive or non-tender ulcers. Primary and post-primary CTB infection progression varies and is influenced by the patient's age, immune system and other similar factors [18]. In our case we also identified prolonged disease progression. Local injury on her right palm was happened five years ago and the skin and skeletal manifestation gradually come up. Young age was associated with stronger immune function which have role to slow down the disease progression [20].

The breakdown of the epidermis surrounding a subcutaneous focus of *Mycobacterium tuberculosis* leads to lesions of endogenous CTB infections. These are classified as orofacial TB or scrofuloderma. When scrofuloderma spreads from lymph nodes to the skin, bones, and joints, it can occasionally cause cutaneous symptoms. A painless, reddish-brown, firm nodule or swelling covering an afflicted lymph node is the initial sign of scrofuloderma. This nodule or swelling indurates and eventually degrades to form a discharging sinus tract filled with watery, purulent, or caseous material. Another name for the earliest manifestation of a subcutaneous, painless nodule is a “cold abscess”. These lesions usually show up in conspicuous lymph node locations, such as the groin, chest wall, neck, and axillae [16]. In this presenting case we could not identify any abnormality that represented scrofuloderma. There was no lymph node enlargement either.

Acute miliary TB, metastatic tuberculous abscess, and lupus vulgaris are examples of hematogenous-spread cutaneous TB. The development of these lesions is the result of *M. tuberculosis* spreading throughout the body from the principal site of infection. Hematogenous cutaneous TB infections are linked to malignancy, disfigurement, and Hodgkin's lymphoma [20]. Lupus vulgaris is the most prevalent type. On the face or neck, lupus vulgaris appears as a cluster of tiny, soft, brown-red papules that eventually combine to form a gelatinous plaque. The buttocks and lower extremities are more frequently affected in the tropics and subtropics. Apple jelly nodules are typically discovered during a diascopy [19].

The diagnosis of TB includes detection, species/complex identification, and drug sensitivity. Absolute and relative criteria were used to establish the diagnosis of CTB. The absolute criteria are as follows: a positive PCR result, guinea pig inoculation and detection of *M. tuberculosis* from tissue culture. The relative criteria consist of the following: a positive tuberculin test, responsiveness to anti-TB medications, active TB found in other organs, finding of AFB on lesions, finding of tuberculous granuloma on histopathological examination, and history taking and clinical manifestations supporting the diagnosis of CTB [7].

Besides a suggestive clinical presentation, the histologic criteria include tuberculoid type granulomas with/without caseous necrosis, with positive tuberculin skin test or TB confirmed in another tissue, and a successful empirical treatment after 1 week. Ziehl–Neelsen staining of the skin lesion is a crucial further examination for the bacteriological study of acid fast bacili (AFB) in order to diagnose CTB. In this presented case series, AFB was discovered. The histological appearance of CTB can vary, but on average, it exhibits granulomas made up of Langhans, plasma, mononuclear, and epithelioid cells, either with or without caseation necrosis [17,18]. Affected individuals with scrofuloderma have fewer developed granulomas [10]. 47.5 % of scrofuloderma patients had a classic histological examination result, according to Kumar et al.; the remaining 52.5 % of patients had a nonspecific result.^23^ The epidermis may appear ulcerative, hyperplastic, or atrophic on lupus vulgaris patient [10]. Several tuberculoid granulomas with caseous necrosis may also be discovered on the superficial dermis of lupus vulgaris [3,10].

Focal necrosis and an abscess were discovered on the initial lesion of acute miliary CTB, and numerous AFB were dispersed around nonspecific inflammatory cells [20]. Tuberculosis verrucosa cutis showed notable epidermal abnormalities, including hyperkeratosis, acanthosis, and papillomatosis. Bacilli are seen at the same time as moderately intense caseous necrosis and tuberculous granulomas are observed in the dermis. Meanwhile primary CTB findings vary depending on when the patient was vaccinated; acute lesions show a necrotizing neutrophilic infiltration with multiple AFB. A subsequent stage is characterized by granuloma organization and a reduction in bacilli. In this case the patient also underwent histopathological examination using tissue biopsy which revealed specific inflammation caused by TB.

TB diagnosis is confirmed by culture and isolation (using Lowenstein-Jensen medium or PCR); however, according to a study by Tirado et al., the first test typically yields negative findings, therefore the second test is frequently needed for this reason. For the diagnosis of TB, the culture has a good sensitivity and specificity. The Ogawa-Kudoh medium is also one of the traditional mycobacteria culture techniques, however the Lowenstein-Jensen medium is the principal one. It can take up to 60 days to see signs of bacterial development, with a range of 14 to 30 days. Molecular methods, phenotypic methods, and biochemical studies are all utilized in the identification of various species. If a non- TB mycobacteriosis, or TB is suspected, a mycobacteria culture is recommended [6,10]. In this case we could not performed the culture test which standardized as a gold standard diagnosis nevertheless we completed histological examination that favor cutaneous TB diagnosis.

The crop with identification of species and drug susceptibility is indicated in the following cases: patients with a history of previous treatment, regardless of how much time has elapsed; in immuno-suppressed patients, especially with HIV; anti-TB treatment failure; research population in high-risk subjects (health professionals, homeless, military, patients admitted to long-term facilities or hospitals that do not take appropriate biosecurity measures), or who are difficult to address for the clinical follow-up (low-income populations). Resistance to treatment can be defined as a decreased in vitro susceptibility of *M. tuberculosis*, when comparing with a wild-type strain (which never had direct contact with anti-TB drugs) [19].

Severe scarring due to CTB lesion may lead to fibrosis, contractures of the joints, and also mutilation [19,20]. Oral, genital, and nasal mucosa can also be involved often as an extension of skin involvement. Rarely, destruction of ear and nose can also be seen. Contractures involving knee, elbow, and wrist joints are other disabling sequelae of CTB. In our case we also observed contracture of her phalanx which caused by severe scarring.

Reduction in range of motion (ROM) in the active or quiet state of the joint is the hallmark of joint contracture, a frequent clinical disease nowadays. Additionally, it typically arises from significant scarring, arthritis, joint damage, or diseases of the central nervous system; however, joint immobility remains the most frequent cause. Joint immobilization is typically utilized as an essential treatment for fractures, dislocations of the joint, and damage to the ligaments, as we currently understand. However, rehabilitation becomes extremely difficult after a lengthy period of immobility to build joint rigidity; even surgery, such as arthroscopic arthrolysis, is difficult to restore full range of motion. In this presenting case we performed surgery to release the contracture. Corresponding treatment of the mechanism of skeletal muscle changes can improve the symptoms of joint contracture, thereby improving the quality of life of patients and benefiting the reasonable distribution of social medical resources.

The standard protocol for treating CTB disease is the same as that for treating pulmonary TB: two months of quadruple-regime therapy (rifampicin, isoniazid, pyrazinamide, and ethambutol) is followed by four months of double therapy (rifampicin and isoniazid). When cutaneous TB is linked to other extra-pulmonary TB symptoms, longer treatments are recommended. A good clinical response usually takes 4–6 weeks to achieve, however treatment failures might happen due to drug-resistant strains that necessitate the use of less effective second-line medications (e.g. capreomycin, kanamycin, ethionamide) [18]. Similar to pulmonary TB, medication toxicity and patient compliance monitoring are crucial. In this case the patient had consumed the anti- TB drugs for three months.

Patients with cutaneous TB who are not immunocompromised have a good prognosis. However, in patients with impaired immune systems or in cases when the organisms are resistant to many drugs, even intensive treatment may not work [13].

## 4 Conclusion

Despite its low occurrence, patients who arrive with unusual skin lesions suggestive of an underlying infectious cause should be evaluated for CTB. For the purpose of preventing the complications, promptly and efficiently diagnosing and treating these extremely serious skin disorders, doctors must possess a high index of suspicion. Complications related to the long-term sequelae of untreated CTB include marked contractures, scarring, and the development of carcinomas and sarcoma. This case study highlights the rarity of the case and significance of a potentially disabling. Early diagnosis results in appropriate treatment, preventing the sequelae events and high levels of patient satisfaction.

## Consent

Written informed consent was obtained from the patient for publication and any accompanying images. A copy of the written consent is available for review by the Editor-in-Chief of this journal on request.

## Ethical approval

Ethical approval for this study (Ethical Committee N° NAC 207) was provided by the Ethical Committee of Dr. Hasan Sadikin General Hospital, Bandung, Indonesia on 15 November 2023.

## Funding

This research did not receive any specific funding.

## Author contribution

Hardisiswo Soedjana: Study concept or design, data collection, data analysis, interpretation, writing the paper.

Betha Egih Riestiano: Study concept or design, data collection, data analysis, interpretation, writing the paper.

Lisa Y. Hasibuan: Study concept or design, data collection, data analysis, interpretation, writing the paper.

Selvy Harianti: Study concept or design, data collection, data analysis, interpretation, writing the paper.

## Guarantor

Hardisiswo Soedjana.

Betha Egih Riestiano.

Lisa Y. Hasibuan.

Selvy Harianti.

## Research registration number

This study does not require registration.

## Declaration of competing interest

The authors declare no conflicts of interest related to this study.
